# Investigation of cases of work-related mental disorders in Brazil: an ecological study

**DOI:** 10.47626/1679-4435-2025-1450

**Published:** 2026-01-02

**Authors:** Benjamim Antônio Pinheiro Vieira, Leticia Barros Ricarte

**Affiliations:** 1 Faculdade de Medicina, Universidade Estadual do Ceará (UECE), Fortaleza, CE, Brasil; 2 Faculdade de Psicologia, Universidade Estadual do Ceará (UECE), Fortaleza, CE, Brasil

**Keywords:** work, mental health, preventive medicine, epidemiology., trabalho, saúde mental, medicina preventiva, epidemiologia.

## Abstract

**Introduction:**

The mental health of Brazilian workers is compromised, and work-related mental disorders are becoming increasingly common. Several factors may be associated with the rise in these cases, among which precarious work stands out as particularly relevant.

**Objectives:**

To describe the epidemiological profile of work-related mental disorders in Brazil between 2015 and 2024.

**Methods:**

This is an ecological study, with a retrospective, descriptive and quantitative approach, which analyzed from the publicly available secondary data.

**Results:**

A total of 21,186 notifications were analyzed. Women accounted for 67% of the cases of work-related mental disorders, showing an upward annual trend. Furthermore, 60% of cases occurred among individuals with completed high school or higher education. In addition, 49% of the diagnoses corresponded to neurotic disorders, stress-related disorders, and somatoform disorders, whereas mood disorder and burnout syndrome accounted for approximately 27% of reported cases. The data also show that 56% of cases of work-related mental disorders progressed to temporary disability. Nonetheless, the study faced limitations due to the high proportion of missing or blank data in the reporting system.

**Conclusions:**

Based on the information presented in this study, it becomes feasible to develop effective preventive strategies to protect workers’ health and safety, as well as to reduce government expenditures.

## Abstract (pt)

**Introdução:**

A saúde mental do trabalhador brasileiro encontra-se prejudicada, e os transtornos mentais relacionados ao trabalho tornam-se cada vez mais comuns. Diversos fatores podem estar associados ao crescimento desses casos, entre os quais a precarização do trabalho constitui um elemento particularmente relevante.

**Objetivos:**

Descrever o perfil epidemiológico dos transtornos mentais relacionados ao trabalho no Brasil entre 2015 e 2024.

**Métodos:**

Trata-se de um estudo ecológico, com abordagem retrospectiva, descritiva e quantitativa, que analisou dados secundários de domínio público.

**Resultados:**

Foram analisadas 21.186 notificações. Observou-se que 67% dos casos de transtornos mentais relacionados ao trabalho corresponderam ao público feminino, com tendência de crescimento anual. Além disso, 60% dos casos envolveram indivíduos com ensino médio ou superior completos. Constatou-se ainda que 49% dos diagnósticos referiam-se a transtorno neurótico, transtornos relacionados ao estresse e transtornos somatoformes, enquanto os transtornos de humor e a síndrome de *burnout* representaram aproximadamente 27% das notificações. Os dados demonstraram que 56% dos casos de transtornos mentais relacionados ao trabalho evoluíram para incapacidade temporária. Destaca-se, entretanto, que o estudo enfrentou limitações decorrentes das taxas de dados ignorados ou em branco no sistema de coleta.

**Conclusões:**

Com base nas informações apresentadas neste estudo, torna-se viável elaborar estratégias preventivas eficazes para resguardar a saúde e a segurança dos trabalhadores, além de reduzir os custos para o governo.

## INTRODUCTION

The term “work” originates from the Latin *tripalium*, a torture device. Although its meaning has changed over time, the association with suffering and constraint persists. Workers’ psychological distress, for instance, may be linked to this antient notion of martyrdom, which remains present today. Furthermore, unstable labor relations, job insecurity, low wages, and precarious working conditions also contribute to this scenario.^1^ Therefore, work-related mental disorders are a reality in Brazil and have become a latent public health issue.

Brazilian workers experience significant impairment of their mental health. The current labor environment exposes workers to stressful conditions, thereby increasing the risk of mental disorders.^2^ Law No. 13,467/2017 reinforces a model of capitalist flexibilization of work that leaves workers with fewer guaranteed rights. The aim of labor volatility has been the reduction of state labor protections. This scenario, combined with poor infrastructure and low wages, contributes to the emergence and progression of work-related mental disorders.^3^

The Brazilian Ministry of Health, through the National Workers’ Health Policy (Política Nacional da Saúde do Trabalhador e da Trabalhadora, PNSTT), states in Article 2 the principles, guidelines, and strategies to be implemented across all levels of the Brazilian Unified Health System (Sistema Único de Saúde, SUS). This regulation aims to ensure the promotion of workers’ health through comprehensive care and individual protection. PNSTT focuses on reducing morbidity and mortality from occupational production processes, thus helping improve workers’ quality of life and well-being in the workplace.^4^

Among the strategies of PNSTT, the establishment of the National Network for Comprehensive Workers’ Health Care (Rede Nacional de Atenção Integral à Saúde do Trabalhador, RENAST) is essential for occupational health surveillance.^5^ RENAST delivers specialized care for workers’ health, helps integrate services within the SUS, and is one of the entities responsible for mandatory reporting of work-related diseases and conditions, through the Notifiable Diseases Information System (Sistema de Informação de Agravos de Notificação, SINAN).^6^

SINAN plays a crucial role in developing epidemiological research. Law No. 6,259 of October 30, 1975, establishes mandatory reporting by physicians, managers, and other health care professionals. This documentation allows for monitoring the labor context in Brazil. Moreover, such data guide the formulation of public or private policies that support workers’ well-being and health.^7^

The Ministry of Health defines work-related mental disorders as manifestations of emotional distress, such as tearfulness, sadness, excessive fear, psychosomatic diseases, agitation, irritability, nervousness, anxiety, tachycardia, sweating, and insecurity, among other symptoms. The Ministry also states that these manifestations arise from causal factors associated with work organization and management or from exposure to certain toxic agents.^8^

It is worth noting that burnout syndrome, depression, suicide attempt, and anxiety, conditions previously overlooked by the Ministry of Health, were added in 2023 to the List of Work-Related Diseases (Lista de Doenças Relacionadas ao Trabalho) as work-related diseases.^9^

The review of Regulatory Standard No. 01, issued on August 27, 2024, through Ordinance No. 1,419, introduces employer’s accountability for worker’s mental health. The standard provides workers with greater autonomy to refuse occupational hazards and encourages companies to include them in its Action Plan, encompassing the identification, assessment, and control of occupational hazards, such as physical, chemical, and biological agents, accident risks, and ergonomic and psychosocial factors.^10^ This process aims to minimize risks that may contribute to the development of mental disorders.

Considering the importance of this topic, the present study aims to analyze the temporal trend of work-related mental disorders, categorized by diagnosed condition, from 2015 to 2024. This study evaluated the number of reported cases according to variables such as sex, notification year, educational level, and diagnosis, as well as the outcome of reported cases. Thus, the article seeks to support the development of initiatives aimed at reducing these occurrences and improving care for affected workers. Furthermore, epidemiological research helps guide the optimization of government expenditures, which was another objective of this study.

## METHODS

This ecological, retrospective, descriptive, and quantitative study analyzed public-domain secondary data obtained via the TABNET platform of Department of Informatics of the Unified Health System (DataSUS). These data are provided by the Ministry of Health through SINAN. Data were collected from February to March 2025 and included all cases of work-related mental disorders in Brazil between 2015 and 2024. The use of secondary data justifies study’s exemption from submission to a Research Ethics Committee.

Data extraction focused on the SINAN category “investigation of work-related mental disorders.” The variables prioritized were “notification year,” “sex,” “educational level,” “specific diagnosis,” and “case outcome.” The retrieved information was tabulated in Google Sheets, where absolute and relative frequencies were calculated using descriptive statistics.

A key limitation of the study is the high amount of missing or data entries in the DataSUS platform, along with inconsistencies in some records. These aspects were taken into account when selecting the variables included.

Furthermore, to substantiate data obtained from SINAN, literature searches were conducted on the SciELO and LILACS databases, using the descriptors “mental health” and “work”, combined with the Boolean operator “AND”. The search yielded 36 articles in SciELO and 223 in LILACS. Inclusion criteria were full-text articles published in the last 5 years and relevant to the study topic. Exclusion criteria included theses, conference abstracts, incomplete papers, and articles not available in full. Additionally, labor legislation and publications by scholars and researchers in the field were reviewed.

## RESULTS

In the present study, 21,186 notifications of work-related mental disorders in Brazil from 2015 to 2024 were examined. The study population was stratified by sex and notification year, as shown in Table 1. An increase in notifications was noted between 2015 and 2019, the period prior to the coronavirus disease 2019 (COVID-19) pandemic. The reduction in 2020 may be attributed to underreporting due to health services overload. After that year, the growth rate resumes its upward trajectory until 2024, when a slight decline is observed. The female group also stands out, showing a high incidence of notifications (approximately 67% of the total).

**Table 1:** Notifications of work-related mental disorders by notification year and sex, Brazil, 2015-2024

Notification year	Missing	Male	Female	Total
Total	1	7.022	14.163	21.186
2015	0	485	704	1.189
2016	0	555	901	1.456
2017	0	729	1.192	1.921
2018	0	605	1.211	1.816
2019	0	785	1.594	2.379
2020	0	441	910	1.351
2021	1	600	1.215	1.816
2022	0	880	1.655	2.535
2023	0	1.073	2.762	3.835
2024	0	869	2.019	2.888

Source: Notifiable Diseases Information System (Sistema de Informação de Agravos de Notificação, SINAN).

With regard to educational level, an association can be observed between mental disorders and higher educational levels, as presented in Table 2. A total of 60% of the reported cases involved individuals who completed high school or higher education, which reflects the complexity of the issue. It is also important to highlight that approximately 19% of notifications contained missing or blank data (Table 2).

**Table 2:** Notifications of work-related mental disorders by notification year and educational level, Brazil, 2015-2024

Notification year	Missing/blank	Illiterate	Incomplete elementary education	Complete elementary education	Incomplete high school education	Complete high school education	Incomplete higher education	Complete higher education	Not applicable	Total
Total	3,963	56	1,111	807	919	6,282	1,364	6,576	108	21,186
2015	234	1	79	64	57	368	91	288	7	1,189
2016	290	6	89	61	65	443	95	398	9	1,456
2017	412	15	114	61	84	594	95	535	11	1,921
2018	326	13	106	64	90	480	118	612	7	1,816
2019	378	5	139	82	98	757	158	753	9	2,379
2020	194	2	92	61	64	421	93	422	2	1,351
2021	331	5	73	66	82	538	105	611	5	1,816
2022	508	3	115	99	82	719	164	826	19	2,535
2023	750	1	159	148	150	1,121	246	1,237	23	3,835
2024	540	5	145	101	147	841	199	894	16	2,888

Source: Notifiable Diseases Information System (Sistema de Informação de Agravos de Notificação, SINAN).

Table 3 shows the distribution of diagnoses of work-related mental disorders in Brazil. A total of 49% of the diagnoses corresponded to neurotic disorders, stress-related disorders, and somatoform disorders. Mood disorders and burnout syndrome comprised roughly 27% of the reported cases. Around 11% of the records were missing, which represented a statistical limitation.

**Table 3:** Diagnosis of specific mental disorders by notification year, Brazil, 2015-2024

Specific diagnosis	2015	2016	2017	2018	2019	2020	2021	2022	2023	2024	Total
Total	1,189	1,456	1,921	1,816	2,379	1,351	1,816	2,535	3,835	2,888	21,186
Other ICD-10s not listed	14	27	35	27	38	62	34	42	86	51	416
Missing ICD-10	82	101	165	400	580	234	183	195	263	177	2,380
Mental disorders due to known psychological conditions (F00-F09)	7	15	10	28	8	5	8	10	6	8	105
Mental and behavioral disorders due to psychoactive substance use (F10-F19)	-	13	10	12	9	6	19	22	9	9	109
Schizophrenia, schizotypal, delusional, and other non-mood disorders (F20-F29)	11	20	26	28	21	26	18	19	15	10	194
Mood [affective] disorders (F30-F39)	292	390	488	364	489	296	346	544	687	491	4,387
Anxiety, dissociative, stress-related, somatoform and other nonpsychotic disorders (F40-F48)	722	812	1,062	812	1,011	595	896	1,192	1,884	1,421	10,407
Behavioral syndromes associated with physiological disturbances and physical factors (F50-F59)	3	3	3	3	11	5	10	14	14	3	69
Disorders of adult personality and behavior (F60-F69)	3	4	6	3	5	1	3	5	16	9	55
Intellectual disabilities (F70-F79)	-	-	1	-	2	1	-	2	-	-	6
Pervasive and specific developmental disorders (F80-F89)	-	-	-	-	1	1	-	-	1	-	3
Behavioral and emotional disorders with onset usually occurring in childhood and adolescence (F90-F98)	-	2	1	-	1	-	1	1	4	2	12
Unspecified mental disorder (F99-F99)	17	20	27	55	87	31	41	98	139	65	580
Symptoms and signs involving cognition, perception, emotional state and behavior (R40-R46)	1	4	14	7	18	14	7	74	73	58	270
Intentional self-harm (X60-X84)	-	1	1	3	7	-	2	3	5	6	28
Persons with potential health hazards related to socioeconomic and psychosocial circumstances (Z55-Z65)	11	20	20	28	30	23	74	59	177	151	593
Evidence of alcohol involvement determined by blood alcohol level (Y90)	-	-	-	-	-	-	-	-	1	-	1
Evidence of alcohol involvement determined by level of intoxication (Y91)	-	-	-	-	-	1	-	-	-	-	1
Work-related conditions (Y96)	2	-	4	-	1	6	23	28	45	12	121
Burnout syndrome (Z73.0)	24	24	48	46	60	44	151	227	410	415	1,449

Source: Notifiable Diseases Information System (Sistema de Informação de Agravos de Notificação, SINAN).

ICD-10 = International Classification of Diseases-10th revision.

Table 4 demonstrates that 56% of cases of work-related mental disorders progressed to temporary disability, highlighting the critical scenario in Brazil. In approximately 97% of cases, recovery was not attained, indicating the persistence of lifelong sequelae for affected workers. It bears noting that 18% of the data were blank or missing, and that approximately 9% of entries in the reporting system were categorized as “others,” without specification.

**Table 4:** Case outcome of work-related mental disorders, Brazil, 2015-2024

Case outcome	2015	2016	2017	2018	2019	2020	2021	2022	2023	2024	Total
Total	1,189	1,456	1,921	1,816	2,379	1,351	1,816	2,535	3,835	2,888	21,186
Missing/blank	210	410	312	351	579	241	346	439	567	362	3,817
Recovery	19	28	61	69	110	43	84	121	135	74	744
Unconfirmed recovery	64	53	96	122	145	144	256	237	533	405	2,055
Temporary disability	794	831	1,212	1,081	1,240	759	894	1,295	2,093	1,683	11,882
Partial permanent disability	24	31	78	70	55	38	51	46	50	52	495
Total permanent disability	4	8	16	22	13	4	4	11	10	8	100
Death due to work-related disease	-	1	1	3	3	1	-	3	2	-	14
Death due to other cause	-	-	1	1	2	-	2	-	-	1	7
Other	74	94	144	97	232	121	179	383	445	303	2,072

Source: Notifiable Diseases Information System (Sistema de Informação de Agravos de Notificação, SINAN).

## DISCUSSION

The results of this study provide insight into the profile of workers with mental disorders in Brazil during the period from 2015 to 2024. The rise in cases, according to the literature, may be explained by the precarious nature of contemporary work.^11^ Existing social inequalities are mirrored in the labor sphere. Long working hours, low wages, and prolonged job searching especially affect Black and Brown women living in peripheral regions.^11^ These barriers reinforce inequalities and support the data presented in Table 1, showing that peripheral populations may also be the most affected by mental disorders.

Furthermore, women, as shown in Table 1, are the group most affected by work-related mental disorders. This finding can be attributed to the double workload experienced by many of them, who, after their workday, still have to manage their household and care for their children and spouse, along with other domestic responsibilities.^12^

It is worth noting that, in 2020, the data presented in Table 1 show a decrease in reported cases. Nevertheless, distancing from the workplace is known to act as a catalyst for anxiety, depression, and other problems.^13^ Among health care workers, the 2020 pandemic also triggered psychological distress, making them one of the groups most affected by work-related disorders during that period.^14^ Therefore, underreporting is a plausible explanation for the decline in reported cases.

Data regarding educational level (Table 2) reveal a clear trend: the higher the educational level, the greater the number of work-related mental disorders. The literature includes numerous studies focusing on this population, especially individuals with higher education. Precarious work affects not only manual workers but also intellectual and highly specialized ones.^15^ Moreover, certain professional categories experience work-family conflict, particularly those with extensive on-site working hours. Pressure from multiple sources contributes to reduced job satisfaction and the affective dimension of commitment.^16^

The family distancing imposed by work increases the likelihood of loneliness and sadness among these professionals, as it entails separation from their family environment. This factor contributes to a higher risk of developing mental disorders.^15^,^16^ The higher rates of these disorders observed among more educated individuals may be related to the limited access of lower-income workers to diagnostic resources, either due to delays in scheduling consultations through the SUS or because individuals do not seek these services.

The findings of the present study indicate that neurotic, stress-related, and somatoform disorders (F40-F48) account for the highest number of notifications. These disorders encompass significant emotional and physical symptoms arising from stress or adaptation difficulties. They include phobias, generalized anxiety disorder, obsessive-compulsive disorder, panic disorder, post-traumatic stress disorder, and somatoform disorders.^17^ The literature describes several evidence-based approaches for managing these conditions, such a cognitive-behavioral therapy, meditation, and yoga, all of which have demonstrated substantial benefits.^18^ However, these practices remain largely inaccessible to populations living in peripheral areas of Brazil, making them a feasible option for only a small proportion of workers.

Mood disorders and burnout syndrome appear with considerable frequency in the data presented in Table 3. These are multifactorial conditions associated with the use of psychoactive substances and various contemporary lifestyles patterns. Due to the complexity of mood disorders, their manifestations, and their treatments, stigma and prejudice often arise, indicating the need for educational actions to address this situation.^19^ This complexity also discourages workers from seeking care, as many do not think treatment is necessary or are unable to identify symptoms. Therefore, the family plays a key role in initiating and maintaining treatment, making its involvement essential.^19^

Hence, individuals with work-related mental disorders encounter multiple barriers to diagnosis, underscoring the need for government interventions to address and eliminate these barriers. Without overcoming this scenario, cases of these disorders tend to increase, an upward trend already evidenced in Tables 1 and 3. Furthermore, these conditions exhibited low recovery rates, highlighting the complexity of the issue. Table 4 shows that only 3% of cases had confirmed full recovery, a concerning figure. This finding reinforces the need for government measures to prevent the deterioration of workers’ mental health. Nonetheless, it should be noted that 2,050 notifications lacked confirmed recovery status, representing a limitation of the data collection platform.

## CONCLUSIONS

The findings of the present study reveal the alarming nature of the mental health situation. These data underscore the importance of PNSTT, together with RENAST and SINAN, in building a database to support studies with this methodological design. Therefore, defining the epidemiological profile of these workers is extremely important for the country. Based on the information presented in this study, it becomes feasible to develop effective preventive strategies to protect workers’ health and safety, as well as to reduce government expenditures.
